# Randomized Trial of Bioceramic Apical Barrier Methods in Necrotic Immature Incisors: Effects on Pain, Extrusion, and Procedure Duration

**DOI:** 10.3390/children12101423

**Published:** 2025-10-21

**Authors:** Yasser Alsayed Tolibah, Nada Bshara, Osama Aljabban, Mohammad Tamer Abbara, Marwan Alhaji, Imad-Addin Almasri, Ziad D. Baghdadi

**Affiliations:** 1 Department of Pediatric Dentistry, Faculty of Dentistry, Damascus University, Damascus P.O. Box 3062, Syria; yasseralsayedtolibah@gmail.com (Y.A.T.); gmmn2012@gmail.com (N.B.); mrwan.2013.55.a@gmail.com (M.A.); 2 Department of Endodontics, Damascus University, Damascus P.O. Box 3062, Syria; dr.ossamaljabban@gmail.com (O.A.); tamerabbara@gmail.com (M.T.A.); 3 Applied Statistics Department, Damascus University, Damascus P.O. Box 3062, Syria; imad_almasri27@yahoo.com; 4 Department of Preventive Dental Sciences, Division of Pediatric Dentistry, University of Manitoba, Winnipeg, MB R3E 0W2, Canada; 5 Centre for Community Oral Health, Dr. Gerald Niznick College of Dentistry, University of Manitoba, P131B, 780 Bannatyne Avenue, Winnipeg, MB R3Y 1P5, Canada; 6 Topsmiles Pediatric Dentistry & Orthodontics, Winnipeg, MB R2M 3A4, Canada

**Keywords:** apical barrier, BioCeramic, BioCeramic extrusion, postoperative pain, treatment duration

## Abstract

**Highlights:**

**What are the main findings?**

- The Single Cone with Bioceramic Sealer (SBS) method yielded the lowest immediate postoperative pain and the shortest procedure duration, rendering it the most child-friendly approach, particularly for less cooperative patients.
- The Bioceramic Putty Apical Plug (BPAP) method demonstrated the lowest extrusion rates, but was associated with higher postoperative pain and significantly longer treatment times

**What are the implications of the main findings?**

- The SBS technique may be advantageous for children who require quicker and less painful treatment sessions, though clinicians should carefully consider the higher risk of material extrusion.
- The BPAP approach may be the method of choice in cases with wide apices or when minimizing extrusion is critical, despite its drawbacks of increased treatment time and patient discomfort.

**Abstract:**

**Objective**: This randomized controlled trial evaluated postoperative pain (PP), bioceramic extrusion, and procedure duration in necrotic immature incisors treated with three apical barrier methods (ABMs): Bioceramic Putty Apical Plug (BPAP), Single Cone with Bioceramic Sealer (SBS), and Bioceramic Putty–Sealer Mixture (BPSM). Case-related factors influencing these outcomes were also examined. **Methods**: Ninety-nine children (8–11 years) with necrotic maxillary incisors and moderate periapical lesions were randomly assigned (1:1:1) to BPAP, SBS, or BPSM groups. Standardized protocols included calcium hydroxide dressing and XP-Endo Finisher irrigation. Pain (VAS) was recorded at 1-, 3-, 7-, and 14-day post-treatment. Extrusion (yes/no) and procedure duration were documented. Regression analyses identified predictors of outcomes. **Results**: At day 1, pain was highest in the BPAP group (mean 3.5) and lowest in the SBS group (mean 1.05; *p* < 0.001). Pain decreased substantially by day 3 and resolved in all groups by day 14. Extrusion was most frequent in SBS (60.6%) and least frequent in BPAP (21.2%; *p* = 0.002). Treatment duration was longest in BPAP (25.8 min) and shortest in SBS (12.6 min; *p* < 0.001). Regression showed that preoperative pain and pulpal diagnosis were the strongest predictors of postoperative pain. Apical size and ABM predicted extrusion, while apical size and child behavior significantly influenced duration. **Conclusions**: The apical barrier method had a significant impact on short-term outcomes. SBS offered reduced pain and shorter chair time but carried a higher risk of extrusion, while BPAP minimized extrusion but caused more pain and required more extended visits. Clinical selection should balance patient comfort, apical anatomy, behavior, and operator expertise. Longer-term outcomes on periapical healing remain to be evaluated.

## 1. Introduction

Dental trauma remains a prevalent issue among children and young adults, with an estimated 25% of school-age children and 33% of adults experiencing trauma to permanent teeth [1]. While these injuries can generally be managed, immature permanent teeth are especially susceptible to complications if treatment is delayed or inadequate, leading to pulp necrosis [2]. Addressing these challenges in immature anterior teeth requires a nuanced approach that balances esthetics, function, and effective management of the root canal system [3]. Notably, the child’s behavior may influence the immediate management of the immature teeth and the long-term treatment outcomes. This fact underscores the importance of considering the child’s behavior as an additional challenge in the endodontic management of immature teeth [4,5].

Apical barrier treatment with calcium silicate cement apical plugs has proven to be an effective treatment for necrotic immature permanent teeth, particularly when root development aligns with stages 2 to 4 of Cvek’s classification [6].

Mineral Trioxide Aggregate (MTA) has served as the gold standard for apical plugs in apexification treatments, demonstrating high radiographic success rates and yielding good clinical and histological outcomes [7,8]. However, MTA has limitations, including tooth discoloration, a prolonged setting time, and handling challenges [9].

Recent advancements have led to the introduction of new calcium silicate-based materials with enhanced composition, handling characteristics, viscosity, and faster setting times, including Biodentine, BioAggregate, and BioCeramics [9].

BioCeramics, a more recent class of hydraulic calcium silicate cements [10], can effectively penetrate dentinal tubules, creating a tight seal while promoting biocompatibility and minimizing inflammation [11,12]. These materials also exhibit antibacterial properties, offering a less invasive treatment option that reduces the need for multiple visits [13,14,15,16]. These materials are available in different viscosities, including TotalFill BioCeramic Sealer, Root Repair Material Paste, Root Repair Material Putty, and Fast Putty [17]. The viscosity raises the question of how these materials are utilized in apical barrier treatments and how the application method of the apical plug influences clinical treatment outcomes.

Several factors have been identified as influencing postoperative pain in closed apices teeth, including preoperative pain, obturation technique, the extent of apical enlargement, and the extrusion of obturation materials [18]. Moreover, it is well established that the use of calcium silicate–based sealers results in less postoperative pain compared to other types of sealers in mature teeth with closed apices [19]. However, the impact of these factors on permanent teeth with open apices remains unknown. Moreover, the use of calcium silicate–based sealers is not yet considered a common practice in the management of immature teeth with open apices, according to recently published literature [9].

In the case of immature permanent teeth indicated for apical barrier treatment, additional apical enlargement is typically not performed. Instead, a conservative preparation of the coronal and middle thirds is carried out to eliminate the reverse taper, thereby facilitating access to the apical third with instruments and irrigation solutions [7]. However, the impact of apex size on the intensity of postoperative pain has not been previously evaluated in studies involving immature teeth.

Notably, the extrusion of obturation materials has been commonly reported in numerous case reports during the treatment of open apices with apical barriers [20,21]. However, no precise studies have been found that correlate the extent of apical width with the extrusion of biomaterials. Moreover, the extrusion of obturation materials has been correlated with postoperative pain and obturation techniques in several recent systematic reviews focusing on endodontic treatment of closed apical teeth [22,23]. However, no studies have specifically addressed these aspects in teeth with open apices.

Children are more susceptible to dental fatigue during prolonged treatment periods associated with traumatic dental injuries [24]. However, the impact of apical barrier treatment visit duration on clinical outcomes remains largely unexplored. Additionally, it is unclear whether this factor is influenced by the obturation technique used or by patient- and tooth-specific factors, such as those proposed previously [25].

This trial directly compared three distinct application methods: the Bioceramic Putty Apical Plugs (BPAP) method, the Single Cone Gutta-percha with BioCeramic Sealer (SBS) method, and the BioCeramic Putty and Sealer Mixture (BPSM) method, and also investigated patient-related factors such as behavior, apical size, and preoperative pain, which may impact treatment outcomes. The null hypothesis (A) stated that the three apical barrier methods were equivalent in terms of postoperative pain. The null hypothesis (B) assumed that the methods would be comparable in terms of the incidence of bioceramic extrusion. Finally, the null hypothesis (C) proposed that the methods would not differ in terms of procedure duration.

## 2. Materials and Methods

### 2.1. Study Design, Settings, and Ethical Approval

This randomized, single-center, single-blinded, controlled trial (RCT) employed a three-arm parallel superiority design with a 1:1:1 allocation ratio. This study was conducted from October 2023 to February 2025 at the Pediatric Dentistry Department, Faculty of Dentistry, Damascus University, Damascus, Syria. This study was conducted in accordance with the ethical principles outlined in the Declaration of Helsinki. The research project was ethically approved by Damascus University (approval number: UDDS-361-13032023/SRC-2654). The project was funded by Damascus University (Funder No. 501100020595) and was registered in the ClinicalTrials.gov registry under ID number NCT06119477 on 22 October 2023. This RCT has been written in accordance with the CONSORT 2010 guidelines [26].

### 2.2. Sample Size Calculation

The sample size was calculated using G* Power 3.1.9.4 (Heinrich-Heine-Universität, Düsseldorf, Germany). It was estimated, based on a previous RCT [27], that the clinical outcomes of apexification and regenerative procedures in managing necrotic immature teeth were compared. A minimum total sample size of 99 teeth (33 in each group) was sufficient for a significance level of 0.05, power of 85%, and 0.34 as effect size f.

### 2.3. Recruitment and Eligibility Criteria

Three hundred patients aged between 8 and 11 years were referred to the Department of Pediatric Dentistry to manage necrotic maxillary anterior immature teeth during the study period. The principal investigator (Y.A.T.) examined the patients and identified those with at least one or more necrotic immature maxillary incisors (central or lateral) presenting with medium-sized periapical lesions (3–5 mm^2^) and apical periodontitis, corresponding to the fourth category of the Periapical Index classification [28] and incisors classified between stages 2 and 4 according to Cvek’s classification with an apical canal diameter of #80 or larger. Preoperative periapical radiographs of the included incisor were taken using the paralleling technique by a digital dental X-ray unit (Hyperlight; Eighteeth, Changzhou, China), which was set at 65 kVp, 2.5 mA, and 0.20 s, and a digital sensor (#1.5 EzSensor Classic; Vatech, Hwaseong-si, Republic of Korea). These periapical radiographs were taken to assess incisor anatomy, periapical lesion size using ImageJ software, Version 1.54k, apex width, and the cause of periapical lesion to determine the included incisors. Patients were considered unsuitable for inclusion if their parents refused participation, if they had heart diseases, immunosuppression, diabetes, or lidocaine sensitivity, or if they exhibited absolutely negative behavior according to the Frankl scale. Incisors were also excluded if they were unsuitable for pulp therapy (non-restorable, had root fractures, or showed severe internal or external resorption), had sever canal curvature (>30 degree), had severe periodontal disease with more than 5 mm of supporting tissue and bone loss, had unsuccessful previous endodontic treatment, or were associated with facial swelling or extraoral cellulitis [7,29].

Therefore, ninety-nine patients were included in the current research. All children participating in the study had their parents sign an informed consent form after being informed about the trial’s details and its therapeutic aspects.

### 2.4. Randomization

With a 1:1:1 allocation ratio, computer-generated random numbers were used to perform the randomization. The allocation sequence was concealed in numbered, opaque, and sealed envelopes that were opened before the commencement of treatment. The sample was randomly allocated into three groups according to the BioCermic apical Barrier method used (BPAP, SCB, or BPSM). Thus, patients were assigned to three groups: Group 1 (BPAP = 33 samples), Group 2 (SCB = 33 samples), and Group 3 (BPSM = 33 samples). If the patient had multiple affected maxillary incisors, treatment was initiated with the tooth responsible for the chief complaint [30]. In cases where none of the involved teeth exhibited symptoms, priority was determined through a simple lottery using sealed papers. The child would select a paper with the number of the tooth to be treated first. A 7-day interval was maintained between the treatment of each tooth to prevent pain from one tooth from influencing the other [31].

### 2.5. Blinding

A double-blinded approach was adopted in the current study, as it was an interventional study; the treating clinician (Y.A.T.) could not be blinded regarding the apical barrier method used to treat the immature necrotic incisors. However, the involved patients and the outcome assessors (M.T.A. and M.A.) were blinded entirely.

### 2.6. Clinical Procedure

#### 2.6.1. Initial Treatment Visit

In a Dedicated Patient Form (DPF), the patient’s gender, age, cause of pulp necrosis (old fracture injuries, old avulsion injuries, caries, recurrent caries, or traumatic orthodontic forces), periapical diagnosis (symptomatic or asymptomatic periodontitis), and preoperative pain level according to the Visual Analog Scale (VAS), pathological mobility, swelling, and fistulas were recorded.

Under buccal and lingual infiltration anesthesia (Huons Lidocaine HCL, Seoul, Republic of Korea) using one carpule “1.8 mL of 2% lidocaine with epinephrine (1:100,000)” and rubber dam (Sanctuary, Ipoh, Malaysia) isolation, all caries and/or previous restorations were removed. Then, the traditional access cavity was refined using an Endo-Z bur (Dentsply Maillefer, Tulsa, OK, USA). The canal orifice was prepared gently using an orifice opener file (Orodeka Ltd., Xincheng, Jining, China). The working length (WL) was determined radiographically using an appropriate K-file, set 1 mm shorter than the radiographic apex, and recorded as a reference in the DPF. Afterward, gradually larger K-file sizes were inserted into the canal until the treating clinician felt the first file to bind (FFB) at the WL, and the next size was noted not to reach that position, where the FFB was recorded as a reference in the DPF of the apex diameter [32]. Afterwards, shaping and debridement of the root canals were achieved by gentle instrumentation with Plex V ORODEKA rotary files (Orodeka Ltd., Xincheng, Jining, China) up to size 25/06 or 35/06 according to the initial canal width of the included teeth [33]. The canal was irrigated with 5 mL of 5.25% NaOCl (Merck, Darmstadt, Germany) (20 mL of 5.25% NaOCl in total). Subsequently, canals were dried using sterile paper points (Gabadent, Foshan, China) and filled with calcium hydroxide dressing (CHD) (Alfares, Damascus, Syria) after mixing with sterile saline solution in a volume ratio (1:1) using a lentulo spiral (Mani, Inc., Utsunomiya, Japan) mounted on a low-speed handpiece. Finally, the incisor was temporarily restored with a glass-ionomer-based restoration (Medifil; Promedica Dental Material Company, GmbH, Neumunster, Germany).

#### 2.6.2. Bioceramic Apical Barrier (BAB) Visit/Visits

After 14 days, patients were asked to rate their pain again before the apical barrier procedures, reflecting the average pain level experienced during the calcium hydroxide dressing period, as measured using the VAS. Additionally, the presence of pathological mobility, swelling, and fistulas was re-evaluated and recorded in the DPF.

The targeted incisor was anesthetized and isolated again, then the temporary filling and CHD were removed. As final irrigation, all canals were filled with 5.25% NaOCl, and then the XP-Endo Finisher file (FKG Dentair, La Chaux-de-Fonds, Switzerland) was inserted 2 mm shorter than the working length at the center of the canal and operated at a speed of 800 rpm and a torque of 1 N·cm. Then, it was passed through the entire perimeter of the canal wall to ensure that it was touching larger areas of the root walls and removing the remaining necrotic pulp and CDH debris. The irrigant was activated for 30 s (2 mL at a time), which was repeated until 20 mL of 5.25% NaOCl was achieved within a total of 5 min. Subsequently, the canals were irrigated with 5 mL of normal saline, then filled with 2 mL of 17% ethylenediaminetetraacetic acid solution (Dentsply Tulsa Dental, Tulsa, OK, USA), which was activated with the XP-Endo Finisher file for 15 s. The procedure was repeated twice [33]. Afterward, the canals were irrigated with 5 mL of normal saline, 5 mL of 5.25% NaOCl, and then dried using sterile paper points.

**Figure 1:**
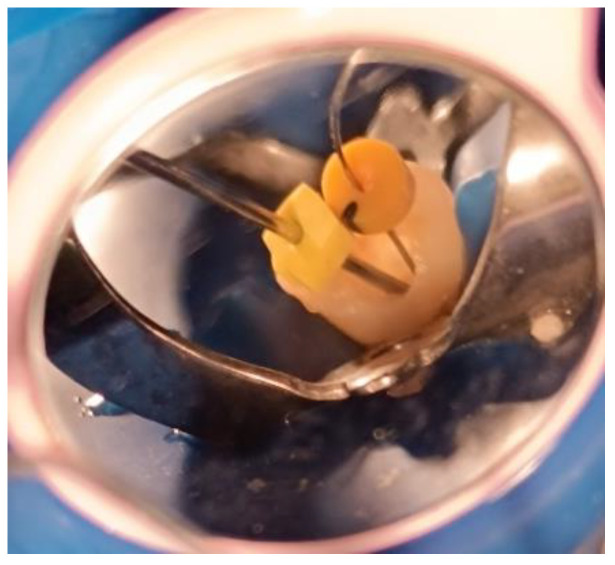
Irrigation procedure used in the present study. Two needles were used: a fine needle to deliver the irrigant to the apical third, and a wider needle positioned closer to the apex to aspirate the irrigant and evacuate it through the chair-mounted surgical suction system.

It is noteworthy that, during irrigant replacement—whether in the initial treatment visit or in the BAB visit—a side-vented irrigation needle (30-gauge) (Sybron Endo, Crop, Orange, CA, USA) was used and positioned 3 mm short of the working length. In addition, a suction needle (22-gauge) was placed 1 mm short of the working length and connected to the chair-mounted surgical suction. This procedure was employed to prevent irrigant extrusion beyond the open apex, as illustrated in Figure 1.

Samples were then divided into three groups according to the BioCeramic apical barrier method adopted using the previously mentioned randomization process. Each method was assigned a specific code and recorded in the patient’s DPF to maintain the assessor’s blinding.

#### 2.6.3. Bioceramic Putty Apical Plugs (BPAP) Group

**Figure 2:**
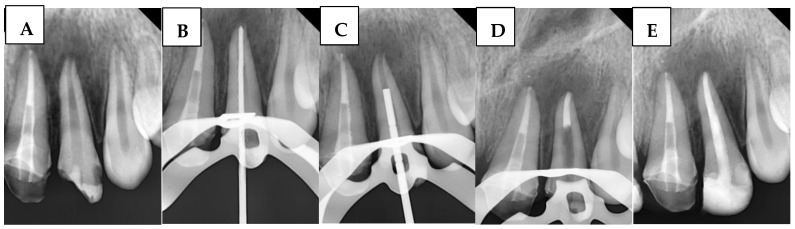
Sequential steps of the procedure in the BPAP group: (**A**) Preoperative periapical radiograph, (**B**) Working length radiograph, (**C**) Plugger fit, (**D**) Apical plug formation, and (**E**) Postoperative radiograph.

A radiograph was first taken with the hand plugger to ensure that it reached 4 mm short of the radiographic apex, as specified by the WL. The BioCeramic Putty (BP) (TotalFill^®^ BC RRM™, FKG Dentaire, Le Crêt-du-Locle, Switzerland) was applied to the apical 4 mm of the canal using a modified cannula [34], with placement adapted using a hand plugger and confirmed radiographically. Any BioCeramic extrusion was recorded at this stage in the DPF. The incisors were then temporized with a cotton pellet and glass ionomer filling. The following day, after isolation and removal of the temporary filling and cotton pellet, the remaining canal space was obturated with gutta-percha and BioCeramic Sealer (BS) (TotalFill^®^ BC Sealer™, FKG Dentaire, Le Crêt-du-Locle, Switzerland) using the cold lateral condensation technique. A periapical radiograph confirmed that the canal was obturated without voids or gaps. Figure 2 illustrates the steps of the procedure in the BPAP group.

#### 2.6.4. Single Cone Gutta-Percha with BioCeramic Sealer (SBS) Group

**Figure 3:**
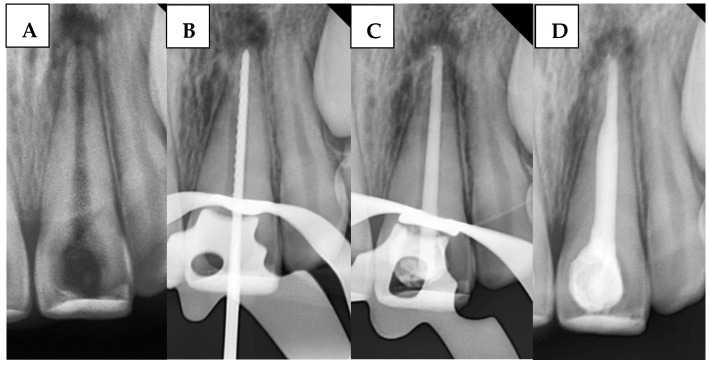
Sequential steps of the procedure in the SBS group: (**A**) Preoperative periapical radiograph, (**B**) Working length radiograph, (**C**) Cone fit, and (**D**) Postoperative radiograph.

A large-sized (#80, taper of 2%) gutta-percha cone (GC) was inserted. The GC was trimmed to fit the canal width as closely as possible without exceeding the WL. The GC was ensured to make tag-back with the apical third of the immature canal and confirmed with a periapical radiograph. Afterward, it was removed, and the immature canal was gently obturated with BS with an adequate amount, and the GC was inserted again, and another apical radiograph was taken to confirm that the canal was properly filled without gaps and voids. Any BioCeramic extrusion was recorded at this stage in the DPF. Figure 3 illustrates the steps of the procedure in the SBS group.

#### 2.6.5. BioCeramic Putty and Sealer Mixture (BPSM) Group

**Figure 4:**
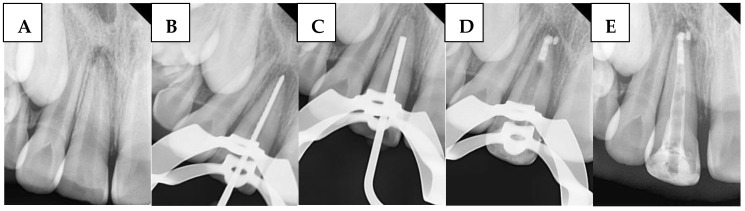
Sequential steps of the procedure in the BPSM group: (**A**) Preoperative periapical radiograph, (**B**) Working length radiograph, (**C**) Plugger fit, (**D**) Apical plug formation, and (**E**) Postoperative radiograph.

A radiograph was first taken with the hand plugger to ensure that it reached 4 mm short of the radiographic apex, as specified by the WL. The immature canal was gently filled with BC. Then, 3 to 5 small pellets (1 mm diameter) of BP were inserted into the canal orifice using a modified cannula [34] and gently plugged with hand pluggers. Finally, an apical radiograph was taken to confirm that the canal was properly filled with a 4 mm BioCeramic apical plug without gaps and voids. Any BioCeramic extrusion was recorded at this stage in the DPF. The incisors were then temporized with a cotton pellet and glass ionomer filling. The following day, after isolation and removal of the temporary filling and cotton pellet, the remaining canal space was obturated with gutta-percha and BS using the cold lateral condensation technique. A periapical radiograph confirmed that the canal was obturated without voids or gaps. Figure 4 illustrates the steps of the procedure in the BPSM group.

Within each group, a digital timer (Simex, Persiceto, Bologna, Italy) was used for each sample to calculate the time needed to form a good canal obturation (without the final restoration), including the two apical barrier visits in the BPAP and BPSM groups and the single apical barrier visit in the SBS group. The required time in minutes was recorded in the DPF.

All Samples within all groups received a suitable final restoration, varying from a resin-bonded restoration to a coronal-radicular restoration, according to the coronal tissue loss.

An external observer, who was not involved in the study, was asked to assess the child’s behavior during all treatment sessions. A single average score was recorded based on the single treatment visit in the SBS group and the full two treatment visits in the BPSM and BPAP groups. The assessment was documented in the DPF according to the Frankl Behavior Rating Scale.

### 2.7. Clinical Follow-Up Periods

Patients were followed up at 1-, 3-, 7-, and 14-day post-treatment and were asked to assess their pain using the VAS. Children indicated their pain levels by marking a point along a 10 cm continuous scale, with endpoints representing no pain and unbearable pain [35]. The pain reported by the patient during each follow-up period was recorded in the DPF.

### 2.8. Outcomes Measurements

#### 2.8.1. The Primary Outcomes Measurements

The DPFs of all patients were reviewed, and the following variables were measured to determine the effect of the three apical barrier methods (BPAP, SBS, and BPSM) on the following:

Postoperative pain at 1, 3, 7, and 14 days after treatment using VAS.

Presence or absence of BioCeramic extrusion at the end of the BAB treatment.

Apical barrier visit/visits duration in minutes, represented by a single BAB visit in the SBS group and both visits combined in the BPAP and BPSM groups.

#### 2.8.2. The Secondary Outcomes Measurements

##### Investigating Factors Affecting Postoperative Pain

The study examines how various factors—including the child’s age, gender, behavior during treatment, apical size, working length, preoperative pain, BioCeramic extrusion, and treatment duration—along with the bioceramic apical barrier method, influence postoperative pain at different follow-up intervals (1, 3, 7, and 14 days).

##### Assessing Factors Influencing BioCeramic Extrusion

The study evaluates the impact of child behavior, apical size, working length, treatment duration, and the bioceramic apical barrier method on the likelihood of material extrusion.

##### Analyzing Factors Affecting Apical Barrier Visit Duration

The study examines the impact of child behavior, apical size, working length, and the selected bioceramic apical barrier method on the total treatment time.

### 2.9. Statistical Analysis

The collected data were tabulated and analyzed using SPSS software (Version 20, IBM SPSS Inc., Chicago, IL, USA). The Kolmogorov–Smirnov test was used to evaluate whether the quantitative measurements showed a normal distribution. Therefore, comparisons between groups regarding postoperative pain (with an abnormal distribution) were performed using the Kruskal–Wallis test, and pairwise comparisons were made using the Mann–Whitney U test. The comparison between groups regarding BioCeramic extrusion (abnormal distribution) was performed using the Chi-square test. The comparison between groups regarding treatment duration (normal distribution) was performed using the One-way ANOVA test, and the pairwise comparisons were performed using the LSD test. Spearman, Mann–Whitney U, and Kruskal–Wallis tests were employed to evaluate the associations among the studied factors and their relationships with the target variables, in accordance with the nature of both dependent and independent variables. Logistic regression was used to identify factors influencing material extrusion. In contrast, multiple linear regression analysis was used to assess the factors influencing post-treatment pain at various time points (1, 3, 7, and 14 days) and the total treatment duration. The level of significance was set at 0.05.

## 3. Results

### 3.1. Descriptive Demographic Characteristics of the Study Sample

**Figure 5:**
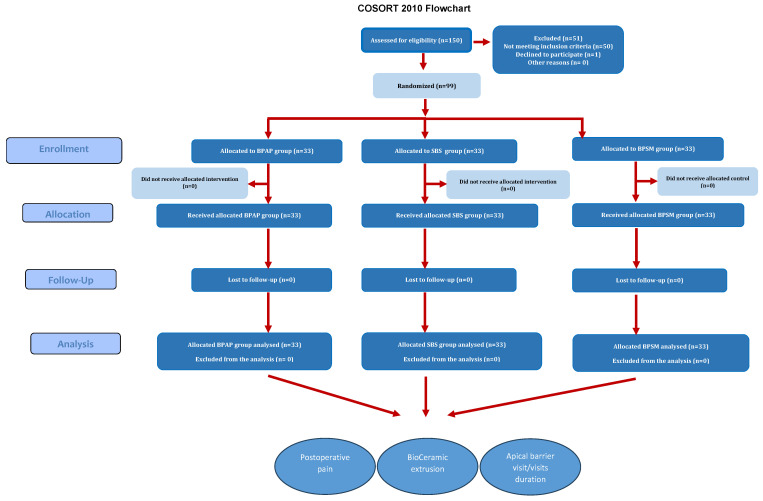
The study flowchart.

The study sample consisted of 99 immature permanent maxillary incisors with pulp necrosis, comprising 59 maxillary incisors in boys and 40 in girls. The overall number of participants was 40 boys and 29 girls. Figure 5 illustrates the flow chart of the study.

Table 1 summarizes the distribution of patients among study groups according to continuous variables: age, working length, apex diameter, preoperative pain level, and pain level before the apical barrier procedure.

Table 2 summarizes the distribution of patients among study groups according to categorical variables: gender, children’s behavior, cause of pulp necrosis, and periapical diagnosis.

As shown in the two previous tables, all uncontrolled variables in the study were randomly distributed across the three groups, with no significant differences in any of the variables (*p* < 0.05), indicating that randomization was effectively achieved. Nevertheless, these variables will be analyzed separately to assess their potential influence on the study’s primary outcomes, namely postoperative pain, BioCeramic extrusion rate, and the apical barrier visit duration.

### 3.2. Analysis of Postoperative Pain and Contributing Factors Across Study Groups

Table 3 presents the comparative results of postoperative pain among the study groups in all time intervals.

The Kruskal–Wallis test revealed statistically significant differences in postoperative pain among the three study groups (BPAP, SBS, and BPSM) only on the first day following treatment. Notably, none of the samples in any of the groups reported any postoperative pain two weeks after treatment.

Table 4 presents the results of pairwise comparisons between the study groups on the one-day postoperative pain.

One-day postoperative pain severity among the groups can be summarized as SBS < BPSM < BPAP.

For the one-day postoperative pain, the Spearman test indicated a negative correlation but not significant regarding age (r = −0.146, *p* = 0.147), positive significant correlation regarding pain before apical barrier procedure level (r = 0.418, *p* < 0.001), positive but not significant correlation regarding working length (r = 0.173, *p* = 0.085), positive but not significant correlation regarding apical size (r = 0.035, *p* = 0.727), and positive significant correlation regarding apical barrier visit duration (r = 0.498, *p* < 0.001). Moreover, the Mann–Whitney test indicated no effects regarding gender (boys/girls) (*p* = 0.361), no effect regarding BioCeramic extrusion (BioCeramic was extruded/BioCeramic was not extruded) (*p* = 0.954), and a significant effect of pulpal diagnosis (symptomatic/asymptomatic) (*p* = 0.002). Finally, the Kruskal–Wallis test revealed no significant effects on child behavior (*p* = 0.843) or the cause of pulp necrosis (*p* = 0.130) on one-day postoperative pain.

Table 5 presents the linear regression results to identify the factor most influencing one-day postoperative pain among the following variables: apical barrier method, pain before the apical barrier procedure, periapical diagnosis, and apical barrier duration.

For the three-day postoperative pain, the Spearman test indicated a positive correlation but not significant regarding age (r = 0.526, *p* = 0.147), positive significant correlation regarding the one day postoperative pain level (r = 0.487, *p* < 0.001), negative but not significant correlation regarding working length (r = −0.012, *p* = 0.903), negative but not significant correlation regarding apical size (r = −0.086, *p* = 0.393), and positive significant correlation regarding apical barrier visit duration (r = 0.241, *p* = 0.016). Moreover, the Mann–Whitney test indicated no effects regarding gender (boys/girls) (*p* = 0.699), no effect regarding BioCeramic extrusion (BioCeramic was extruded/BioCeramic was not extruded) (*p* = 0.721), and a significant effect of pulpal diagnosis (symptomatic/asymptomatic) (*p* = 0.011). Finally, the Kruskal–Wallis test revealed no significant effects on child behavior (*p* = 0.564) or the cause of pulp necrosis (*p* = 0.470) on three-day postoperative pain.

Table 6 presents the linear regression results to identify the factor most influencing three-day postoperative pain among the following variables: apical barrier method, presence of one-day postoperative pain, periapical diagnosis, and apical barrier duration.

For the one-week postoperative pain, the Spearman test indicated a positive correlation but not significant regarding age (r = 0.008, *p* = 0.937), positive but not significant correlation regarding the three-day postoperative pain level (r = 0.0122, *p* = 0.876), positive but not significant correlation regarding working length (r = 0.134, *p* = 0.183), negative but not significant correlation regarding apical size (r = −0.068, *p* = 0.504), positive but not significant correlation regarding apical barrier visit duration (r = 0.027, *p* = 0.786). Moreover, the Mann–Whitney test indicated no effects regarding gender (boys/girls) (*p* = 0.549), no effect regarding BioCeramic extrusion (BioCeramic was extruded/BioCeramic was not extruded) (*p* = 0.807), and no effect of pulpal diagnosis (symptomatic/asymptomatic) (*p* = 0.71). Finally, the Kruskal–Wallis test revealed no significant effects on child behavior (*p* = 0.528) or the cause of pulp necrosis (*p* = 0.838) on one-week postoperative pain.

One week postoperatively, the multiple linear regression analysis revealed that the only factor influencing pain intensity was the use of the apical barrier method (β = 0.054, CI: 0.002–0.093, *p* = 0.021).

### 3.3. Analysis of BioCeramic Extrusion and Contributing Factors Across Study Groups

Table 7 presents the comparison results between the study groups regarding the BioCeramic extrusion according to the evaluated teeth.

The chi-square test showed a significant difference between the groups (*p* = 0.002). Pairwise comparisons using Fisher’s Exact Test were conducted to assess differences in the presence of BioCeramic extrusion among the three study groups. A statistically significant difference was found between the BPAP and SBS groups (*p* = 0.001), indicating a higher incidence of BioCeramic extrusion in the SBS group. However, no significant difference was detected between the BPAP and BPSM groups (*p* = 0.287). The comparison between the SBS and BPSM groups approached statistical significance but did not reach it (*p* = 0.080). BioCeramic extrusion among the groups can be summarized as SBS − BPSM > BPAP.

For the BioCeramic extrusion, the Mann–Whitney test indicated an effect regarding apical size (*p* = 0.003), no effect regarding the working length (*p* = 0.977), and no effect regarding the apical barrier visit duration (*p* = 0.409). Finally, the Chi-Square test indicated no effect on child behavior (*p* = 0.487) regarding the BioCeramic extrusion.

Table 8 presents the binary logistic regression coefficients analyzing the factors influencing BioCeramic extrusion, specifically the apical barrier method and apical size.

### 3.4. Analysis of Apical Barrier Visit Duration and Contributing Factors Across Study Groups

Table 9 presents the comparison results between the study groups regarding the apical barrier visit duration minutes among groups.

The One-way ANOVA test revealed statistically significant differences in apical barrier procedure duration and postoperative pain among the three study groups (BPAP, SBS, and BPSM) (*p* < 0.001).

Table 10 presents the results of pairwise comparisons between the study groups regarding the duration of the apical barrier procedure.

Apical barrier duration procedure among the groups can be summarized as SBS < BPSM < BPAP.

For the apical barrier procedure duration, the Spearman test revealed a positive but nonsignificant correlation with working length (r = 0.133, *p* = 0.187) and a positive, significant correlation with apical size (r = 0.289, *p* = 0.003). Finally, the Kruskal–Wallis test revealed significant effects regarding the apical barrier method (*p* < 0.001) and child behavior (*p* = 0.007) on the duration of the apical barrier procedure. Specifically, children with negative behavior required a more extended treatment duration than those with upbeat and definitely positive behavior, according to the Frankl scale.

Table 11 presents the linear regression results to identify the factor most influencing the apical barrier visit duration among the following variables: apical size, apical barrier method, and child behavior.

## 4. Discussion

In light of the increasing prevalence of necrotic immature permanent teeth requiring endodontic management in children aged 6–12 years [36], the significance of this study lies in several key aspects. First, it represents the first randomized controlled clinical trial to evaluate the impact of different bioceramic application methods on critical clinical outcomes, including postoperative pain, treatment duration, and the incidence of obturation material extrusion, with the aim of identifying the most effective approach for pediatric patients. Second, it explores the influence of various contributing factors that cannot be fully standardized in a randomized controlled setting—such as apical size, working length, etiology of pulpal necrosis, preoperative symptoms, and children’s behavior—in addition to the selected apical barrier method, all of which collectively shape the overall treatment outcomes.

The use of bioceramics in the presented study has been investigated through three distinct methods. For instance, Sockalingam et al. [14] and Ghaly et al. [29] recommended the application of BioCeramic Putty as an apical plug. Rencher et al. [37] demonstrated that BioCeramic putty and BioCeramic sealer were employed for retrograde filling after apicoectomy. Additionally, BioCeramic sealer use in conjunction with the single-cone method was based on manufacturer guidelines, with reports of high success rates in managing large periapical lesions in closed-apex anterior teeth [38]. However, this method requires modifications for open-apex cases, as the gutta-percha cone cannot achieve tug-back at the open apex. Instead, tug-back is accomplished at the apical third of the root, where the BioCeramic sealer’s high flowability seals the gap between the gutta-percha cone and apical walls.

The present findings indicate a direct association between the apical barrier method using bioceramics and postoperative pain, as the different methods employed demonstrated varying effects on pain intensity. Accordingly, the null hypothesis (A) was rejected. These results highlight the importance of considering the treatment method in patients who are particularly sensitive to endodontic procedures, to minimize discomfort in children during sessions involving apical barrier formation. The regression results indicate that the apical barrier method continued to exert a significant influence on the outcomes even one week after treatment.

The most clinically significant finding was the substantial difference in immediate postoperative pain between treatment groups. On the first day post-treatment, pain levels varied dramatically: BPAP showed the highest pain, followed by BPSM, while SBS demonstrated the lowest pain levels.

This pain hierarchy (SBS < BPSM < BPAP) suggests that the apical barrier method employed in the apical barrier approach may play a role in influencing postoperative pain when managing necrotic immature permanent teeth. The SBS method is less traumatic to the periapical tissues, potentially contributing to reduced pain levels. The significantly lower pain reported in the SBS group may be attributed to the less invasive nature of the single cone obturation technique, which avoids the use of hand pluggers for apical adaptation. In contrast, both the BPSM and BPAP groups involved the use of hand pluggers to adapt the bioceramic material in the apical third, potentially causing additional trauma to the periodontal ligament and contributing to increased postoperative discomfort. This finding is somewhat in agreement with the study by Ruiz-Cano et al. [39], which showed a tendency for lower postoperative pain—though not statistically significant—when comparing warm vertical compaction to the single cone technique, both using a bioceramic sealer, in the treatment of mature permanent teeth in adult patients after 24 h of the treatment.

By day three, pain levels had substantially decreased across all groups, with no significant differences, and by two weeks, all patients were pain-free. This temporal pattern is consistent with recent studies on bioceramic materials, which demonstrate rapid healing and reduced tissue irritation [7,9].

The reduced postoperative pain observed within groups in this study may be attributed to their distinct immunomodulatory and bioactive properties. Calcium-silicate-based formulations promote a healing response dominated by M2 macrophages rather than the pro-inflammatory M1 phenotype. This polarization leads to downregulation of key pro-inflammatory cytokines, including TNF-α, IL-1β, and IL-6, which are directly associated with periapical inflammation and postoperative discomfort [40,41]. Both in vitro and in vivo studies have consistently demonstrated that BioCeramics induce a minimal cytotoxic and inflammatory response [42,43], likely contributing to the lower incidence of pain following treatment.

Several factors influenced postoperative pain intensity in the current sample. A significant positive correlation was found between preoperative pain and postoperative pain, as well as with the periapical diagnosis, indicating that patients with pre-existing discomfort were more likely to experience post-treatment pain. Notably, regression analysis revealed that these two factors had a greater impact on postoperative pain than other variables (apical barrier visit duration and apical barrier method) on both the first day and three days after treatment. Based on these findings, it is recommended to adopt the less painful method (SBS) when managing cases with acute periapical inflammation and high levels of preoperative pain during apical barrier procedures. Similar evidence in mature teeth supports that a longer duration of preoperative pain significantly predicts increased postoperative discomfort [44].

Longer procedure duration correlated with increased postoperative pain, suggesting that extended treatment times may contribute to tissue trauma and subsequent discomfort. While this specific correlation has not been extensively detailed in previous studies, earlier systematic reviews have primarily focused on comparing postoperative pain between single-visit and multiple-visit endodontic treatments, rather than examining the impact of procedure duration per se on pain outcomes [45]. Therefore, it is recommended that studies focusing on apical barrier methods consider both the visit duration and the presence of preoperative pain in acute cases, as these variables may significantly influence postoperative pain outcomes.

It is worth noting that, among the treated samples in this study across all groups, larger apical diameters were not associated with increased postoperative pain. This finding may be logical, as the immature apices were not subjected to any mechanical enlargement. A recent well-designed study has suggested that the degree of apical preparation significantly influences the severity of postoperative pain [31]. Regarding irrigant extrusion, although a wider apical diameter is generally associated with an increased risk of irrigant extrusion beyond the apex [46], the irrigation protocol used in this study—which involved one side vented irrigation needle to deliver the solution and another tip connected to a saliva ejector for simultaneous aspiration—seemed effective in preventing irrigant extrusion, and therefore did not contribute to increased postoperative discomfort.

The present findings indicate a direct association between the apical barrier method using bioceramics and BioCeramic extrusion, as the BioCeramic extrusion rates among groups revealed important method considerations. Accordingly, the null hypothesis (B) was rejected. The BPAP group showed the lowest extrusion rate, while the SBS and BPSM groups demonstrated higher rates. This finding suggests that the putty consistency in BPAP may provide better control during placement, reducing the likelihood of material overflow beyond the apex. On the other hand, the higher extrusion rates in the SBS and BPSM groups may be related to the flowable nature of sealers and the technique-sensitive nature of these procedures.

Sealers show variable extrusion rates depending on their consistency and application method; Specifically, the bioceramic sealer demonstrated a high extrusion rate, which can be attributed to its excessive flowability and high fluidity [47,48].

It is noteworthy that, within the scope of this study, the BioCeramic extrusion in all study groups did not result in increased postoperative pain. This finding is consistent with previous studies that investigated the impact of sealer extrusion in both mature [47,49] and cases with open apices [20,50]. In line with the existing literature [49], and considering the context of treating nonvital immature permanent teeth, we do not recommend any apical extrusion of the obturation material, as the long-term fate of extruded BioCeramic materials remains unclear—particularly regarding its effect on periapical healing, bone regeneration, and the density of newly formed bone.

Apical size significantly influenced BioCeramic extrusion risk, with larger apical openings predisposing to material overflow. This correlation highlights the importance of carefully selecting cases and modifying methods based on anatomical considerations [51].

It is noteworthy that, based on the logistic regression results regarding the factors influencing BioCeramic extrusion, the apical barrier method itself proved to be more important than the size of the apical foramen. This highlights the crucial role of proper practice and training in each method, as well as the need to adopt the less extrusion-prone method (BPAP) to minimize the risk of sealer extrusion, particularly in wider apical foramina.

The present findings indicate a direct association between the apical barrier method using bioceramics and apical barrier visit duration, as the duration among groups revealed important method considerations. Accordingly, the null hypothesis (C) was rejected.

Samples in the SBS group required the shortest time, followed by BPSM and BPAP, taking the longest. The efficiency advantage of the SBS method may be related to the simplified single-cone obturation procedure compared to the more complex putty manipulation required in other methods. Moreover, it is noteworthy that the SBS method did not require an additional session for filling the remaining portion of the canal. Once the gutta-percha was severed, the final restoration could be accomplished immediately in routine cases that did not necessitate a post-and-core build-up.

Recent clinical reviews confirm that single cone obturation with bioceramic sealers represents a simplified approach that has gained widespread acceptance [52]. Contemporary studies demonstrate that the single cone technique can be used with a bioceramic sealer, which makes obturation faster [53].

Longer procedure times correlated with larger apical sizes, likely due to increased difficulty in achieving adequate seal with wider apical openings. This study also demonstrated that children with definitely negative and negative behavior required longer treatment times regardless of the apical barrier method used. This finding aligns with previous systematic reviews that confirmed a relationship between treatment session duration and child behavior [54]. The use of the SBS method may be advisable in cases involving uncooperative pediatric patients who require apical barrier procedures.

It is likely that the use of the modified cannula [34] for the BioCeramic putty in the BPAP and BPSM groups, combined with the application of ready-to-use premixed BioCeramic in the SBS and BPSM groups, contributed to reducing the influence of WL on the overall treatment duration.

It was observed that the size of the apical foramen and child behavior had a greater impact on the duration of apical barrier visit procedures than the apical barrier method itself. Therefore, these factors should be carefully considered, favoring faster methods in uncooperative children, along with an accurate assessment of the immature permanent tooth anatomy, when selecting the most appropriate bioceramic apical barrier method.

These findings suggest that the SBS method may be advantageous for children who require faster, less painful treatment sessions, although clinicians must weigh the increased risk of extrusion. Conversely, BPAP may be preferred in cases with wide apices or when extrusion must be avoided, despite its drawbacks in time and discomfort. Clinical decision-making should therefore integrate patient cooperation, apex morphology, and operator skill rather than relying on a single technique.

While this study focused on immediate outcomes, it is essential to contextualize these findings within long-term success data from recent literature, which demonstrates high success rates with bioceramic materials in pediatric endodontics across different methods [7,29]. As a next step, a dedicated report will address the long-term effects of the three tested methods on the healing of periapical lesions in immature permanent teeth, as well as the impact of sealer extrusion on the healing process.

One of the limitations of this study is the inability to apply all methods to the same child, as it was not feasible to recruit children with three upper incisors in comparable conditions for these treatments. Additionally, operator blinding was not feasible, and extrusion was recorded only as present/absent, not quantitatively. Cost analysis was also not addressed. Future studies should adopt standardized extrusion measurement and incorporate economic considerations to inform evidence-based guidelines more effectively.

While the present trial highlights short-term differences in pain, extrusion, and treatment duration across bioceramic apical barrier methods, these findings should also be interpreted in the broader context of regenerative endodontics. Advances in pulp revascularization and tissue engineering increasingly emphasize biologic healing and root maturation as ultimate goals. The predictable control of extrusion and minimization of postoperative discomfort observed in this study provide a clinical foundation upon which regenerative protocols can build. Long-term evaluations comparing apical barrier techniques with regenerative approaches will be essential to determine not only periapical healing but also the potential for continued root development, dentin thickness, and preservation of esthetics in growing children.

## 5. Conclusions

Bioceramic apical barrier methods have been shown to influence short-term clinical outcomes in immature necrotic incisors. While SBS offers efficiency and comfort, BPAP provides greater control over the extrusion process. The optimal method should be tailored to clinical anatomy and patient cooperation, with further research needed to confirm long-term outcomes.

## Figures and Tables

**Table 1:** Descriptive demographic characteristics of the continuous variables among the study groups.

Variables	Group	Sample Number	Mean ± Standard Deviation	Range	F-Value	* *p*-Value
Children Age (in years)	BPAP	33	10 ± 2	7–13	2.8	0.068
	SBS	33	10 ± 2	7–12		
	BPSM	33	11 ± 1	8–13		
	Total	99	10 ± 2	7–13	-	-
Working Length (in mm)	BPAP	33	22 ± 2	19–25	2.5	0.089
	SBS	33	21 ± 2	16–26		
	BPSM	33	20 ± 3	16–26		
	Total	99	21 ± 2	16–26	-	-
Apical Size	BPAP	33	1 ± 0.5	0.8–1.5	1.8	0.171
	SBS	33	1 ± 0.5	0.8–1.4		
	BPSM	33	1 ± 0.5	0.8–1.5		
	Total	99	1 ± 0.5	0.8–1.5	-	-
Preoperative Pain Level	BPAP	33	3.1 ± 3.0	0–8	1.7	0.192
	SBS	33	3.9 ± 2.9	0–9		
	BPSM	33	2.2 ± 2.8	0–8		
	Total	99	3.1 ± 2.9	0–9	-	-
Pain before Apical Barrier Procedure Level	BPAP	33	1.4 ± 1.6	0–5	1.1	0.36
	SBS	33	2.2 ± 2.4	0–7		
	BPSM	33	1.4 ± 2.2	0–8		
	Total	99	1.7 ± 2.1	0–8	-	-

BPAP: BioCeramic Putty Apical Plug. SBS: Single Cone Gutta-percha with BioCeramic Sealer. BPSM: BioCeramic Putty and Sealer Mixture. * One-way ANOVA test.

**Table 2:** Descriptive demographic characteristics of the categorial variables among the study groups.

Variable		Group			Chi-Square Value	* *p*-Value
		**BPAP**	**SBS**	**BPSM**		
**Gender**	**Boys**	**19**	19	21	1.1	0.592
	**Girls**	14	14	12		
**Child Behavior According to Frankel**	**Negative**	10	8	8	0.6	0.963
	**Positive**	9	12	12		
	**Definitely Positive**	10	9	9		
**Pulp Necrosis Cause**	**Old fracture** **Injuries**	18	15	19	1.6	0.99
	**Old avulsion Injuries**	3	5	3		
	**Caries**	8	9	7		
	**Recurrent Caries**	4	4	3		
	**Traumatic Orthodontic Forces**	0	0	1		
**Periapical Diagnosis**	**Symptomatic**	13	10	9	1.2	0.55
	**Asymptomatic**	20	23	24		

BPAP: BioCeramic Putty Apical Plug. SBS: Single Cone Gutta-percha with BioCeramic Sealer. BPSM: BioCeramic Putty and Sealer Mixture. * Chi-square test.

**Table 3:** Descriptive statistics of postoperative pain according to the adopted apical barrier method and the significance of the difference among the groups.

Time Intervals		BPAP	SBS	BPSM	* *p*-Value
**One-day Postoperative Pain**	Mean	3.5	1.1	2.2	<0.001 ^
	Standard Deviation	1.4	1.3	1.5	
	Range	0–6	0–3	0–5	
**Three-day Postoperative Pain**	Mean	0.6	0.1	0.3	0.058
	Standard Deviation	0.8	0.3	0.9	
	Range	0–2	0–1	0–3	
**One-week Postoperative Pain**	Mean	0	0	0.05	0.368
	Standard Deviation	0.0	0.0	0.2	
	Range	0	0	0–1	
**Two-week Postoperative Pain**	Mean	0	0	0.0	1.000
	Standard Deviation	0.0	0.0	0.0	
	Range	0	0	0	

BPAP: BioCeramic Putty Apical Plug. SBS: Single Cone Gutta-percha with BioCeramic Sealer. BPSM: BioCeramic Putty and Sealer Mixture. *: Kruskal–Wallis test. ^: Significant at the 0.05 level.

**Table 4:** The pairwise comparisons between the study groups regarding pain intensity on the first postoperative day.

Group	Mean Difference	95% Confidence Interval for Difference		* *p*-Value
		Lower	Upper	
**SBS vs. BPAP**	−2.4	−3.5	−1.4	<0.001 ^^^
**SBS vs. BPSM**	−1.1	−2.1	−0.1	0.035 ^^^
**BPAP vs. BPSM**	1.3	0.2	2.4	0.014 ^^^

BPAP: BioCeramic Putty Apical Plug. SBS: Single Cone Gutta-percha with BioCeramic Sealer. BPSM: BioCeramic Putty and Sealer Mixture. *: Bonferroni test. ^^^: Significant at the 0.05 level.

**Table 5:** Linear regression coefficients for the analysis of factors influencing the one-day postoperative pain.

	Factor	β (Coefficient)	Standard Error	95% Confidence Interval for Difference		*p*-Value ^a^	Variance Inflation Factor
				Lower	Upper		
**One-day Postoperative Pain**	**Apical barrier method**	0.24	0.10	−0.01	0.39	0.017 *	1.09
	Periapical diagnosis	1.08	0.29	−1.52	0.73	<0.001 *	1.09
	Pain before apical barrier procedure	1.06	0.21	−0.05	0.29	<0.001 *	1.06
	Apical barrier visit duration	0.11	0.02	0.07	0.14	<0.001 *	1.16

^a^ Multiple Linear Regression Analysis. * Significant difference.

**Table 6:** Linear regression coefficients for the analysis of factors influencing the three-day postoperative pain.

	Factor	β (Coefficient)	Standard Error	95% Confidence Interval for Difference		*p*-Value ^a^	Variance Inflation Factor
				Lower	Upper		
**Three-day Postoperative Pain**	**Apical barrier method**	0.12	0.06	0.01	0.22	0.036 *	1.09
	Pulpal diagnosis	0.34	0.17	−0.69	0.57	0.047 *	1.09
	One-day Postoperative Pain	0.30	0.12	−0.04	0.15	0.033 *	1.06
	Apical barrier visit duration	0.03	0.01	0.01	0.04	0.008 *	1.15

^a^ Multiple Linear Regression Analysis. * Significant difference.

**Table 7:** The descriptive statistics of BioCeramic extrusion according to the evaluated teeth in each study group, along with the statistical significance of differences between the groups.

Group		No BioCeramic Extrusion	BioCeramic Extrusion	* *p*-Value
**BPAP**	Count	26	7	0.002 ^
	Percentage	78.79%	21.21%	
**SBS**	Count	13	20	
	Percentage	39.40%	60.60%	
**BPSM**	Count	15	18	
	Percentage	45.45%	54.55%	

BPAP: BioCeramic Putty Apical Plug. SBS: Single Cone Gutta-percha with BioCeramic Sealer. BPSM: BioCeramic Putty and Sealer Mixture. *: Chi-Square test. ^: Significant at the 0.05 level.

**Table 8:** Binary logistic regression coefficients for the analysis of factors influencing obturation extrusion.

Factor	Standard Error	Wald	95% Confidence Interval for Difference		*p*-Value ^a^	Odds Ratio (OR)
			Lower	Upper		
**Apical Barrier method**	0.15	4.38	0.5	1.39	0.006 *	1.44
**Apical Size**	0.36	2.56	0.2	1.73	0.029 *	1.27

^a^ Logistic Regression Analysis.* Significant difference.

**Table 9:** The descriptive statistics of the apical barrier visit duration in minutes across the study groups, with the statistical significance of differences between the groups.

	Groups	BPAP	SBS	BSPM	* *p*-Value
**Apical Barrier Procedure Duration (in minutes)**	Mean	25.75	12.6	17	<0.001 ^
	Standard Deviation	5.54	6.13	6.91	
	Range	16–36	3–22	4–30	

BPAP: BioCeramic Putty Apical Plug. SBS: Single Cone Gutta-percha with BioCeramic Sealer. BPSM: BioCeramic Putty and Sealer Mixture. *: One-way ANOVA test. ^: Significant at the 0.05 level.

**Table 10:** The pairwise comparisons of apical barrier procedure duration between the study groups.

Groups	Mean difference	95% Confidence Interval for Difference		* *p*-Value
		Lower	Upper	
**SBS vs. BPAP**	**−13.1**	−17.1	−9.2	<0.001 ^
**SBS vs. BPSM**	−4.4	−8.3	−0.5	0.029 ^
**BPAP vs. BPSM**	8.7	4.8	12.6	<0.001 ^

BPAP: BioCeramic Putty Apical Plug. SBS: Single Cone Gutta-percha with BioCeramic Sealer. BPSM: BioCeramic Putty and Sealer Mixture. *: LSD test. ^: Significant at the 0.05 level.

**Table 11:** Linear regression coefficients for the analysis of factors influencing the apical barrier visit duration.

	Factor	β (Coefficient)	Standard Error	95% Confidence Interval for Difference		*p*-Value ^a^	Variance Inflation Factor
				Upper	Lower		
**Apical Barrier Visit Duration**	**Apical barrier method**	0.95	0.33	−1.26	0.9	0.004 *	1.07
	**Child behavior**	3.61	0.95	1.24	5.12	<0.001 *	1.04
	**Apical size**	7.68	1.95	2.68	10.55	<0.001 *	1.05

^a^ Multiple Linear Regression Analysis. * Significant difference.

## Data Availability

The data are available upon request from the authors due to ethical reasons.
